# Digital Mental Health Interventions for Older Adults: A Meta-Analysis of Randomized Controlled Trials

**DOI:** 10.1093/geroni/igaf122.3474

**Published:** 2025-12-31

**Authors:** Yahui Wang, Dannii Yeung

**Affiliations:** City University of Hong Kong, Hong Kong, China (People’s Republic); City University of Hong Kong, Hong Kong, China (People’s Republic)

## Abstract

With growing mental health concerns among older adults and limited access to traditional services, digital interventions may offer a scalable solution. However, evidence regarding their effectiveness across different mental health outcomes remains unclear. Using multilevel modelling, a meta-analysis of randomized controlled trials examining digital interventions for older adults was conducted. Forty studies comprising 5,148 participants were analysed. Primary outcomes were depression and anxiety, with secondary outcomes including cognitive functioning, loneliness, and quality of life. Compared to control conditions, digital interventions demonstrated significant beneficial effects for depression (g = -0.28, 95% CI [-0.43, -0.13], p < .001) and anxiety (g = -0.22, 95% CI [-0.45, 0.00], p = .05). Secondary outcomes showed effects for cognitive functioning (g = 0.35, 95% CI [0.06, 0.64], p < .05) and loneliness (g = -0.29, 95% CI [-0.50, -0.08], p < .01), with effects on quality of life approaching significance (g = 0.22, 95% CI [-0.02, 0.46], p = .07). Moderator analyses for depression revealed larger effects for interventions employing guided human support and for smartphone-delivered interventions compared to unguided or internet-based approaches. Digital interventions appear effective for addressing mental health concerns in older adults, depression and anxiety, improving cognitive functioning and loneliness. The significant heterogeneity suggests that effectiveness varies based on intervention delivery method and the inclusion of human guidance. These findings not only highlight the potential of digital mental health tools for expanding psychotherapy options for older adults, but also suggest that personalized intervention addressing the individual needs can maximize the intervention outcomes.

